# High Spatial-Resolution Red Tide Detection in the Southern Coast of Korea Using U-Net from PlanetScope Imagery

**DOI:** 10.3390/s21134447

**Published:** 2021-06-29

**Authors:** Jisun Shin, Young-Heon Jo, Joo-Hyung Ryu, Boo-Keun Khim, Soo Mee Kim

**Affiliations:** 1 BK21 School of Earth and Environmental Systems, Pusan National University, Busan 46241, Korea; sjs1008@pusan.ac.kr (J.S.); joyoung@pusan.ac.kr (Y.-H.J.); bkkhim@pusan.ac.kr (B.-K.K.); 2 Korea Ocean Satellite Center, Korea Institute of Ocean Science and Technology (KIOST), Busan 49111, Korea; jhryu@kiost.ac.kr; 3 Maritime ICT R&D Center, Korea Institute of Ocean Science and Technology (KIOST), Busan 49111, Korea

**Keywords:** *Margalefidinium polykrikoides*, PlanetScope, southern coast of Korea, convolutional neural network, U-Net

## Abstract

Red tides caused by *Margalefidinium polykrikoides* occur continuously along the southern coast of Korea, where there are many aquaculture cages, and therefore, prompt monitoring of bloom water is required to prevent considerable damage. Satellite-based ocean-color sensors are widely used for detecting red tide blooms, but their low spatial resolution restricts coastal observations. Contrarily, terrestrial sensors with a high spatial resolution are good candidate sensors, despite the lack of spectral resolution and bands for red tide detection. In this study, we developed a U-Net deep learning model for detecting *M. polykrikoides* blooms along the southern coast of Korea from PlanetScope imagery with a high spatial resolution of 3 m. The U-Net model was trained with four different datasets that were constructed with randomly or non-randomly chosen patches consisting of different ratios of red tide and non-red tide pixels. The qualitative and quantitative assessments of the conventional red tide index (RTI) and four U-Net models suggest that the U-Net model, which was trained with a dataset of non-randomly chosen patches including non-red tide patches, outperformed RTI in terms of sensitivity, precision, and F-measure level, accounting for an increase of 19.84%, 44.84%, and 28.52%, respectively. The *M. polykrikoides* map derived from U-Net provides the most reasonable red tide patterns in all water areas. Combining high spatial resolution images and deep learning approaches represents a good solution for the monitoring of red tides over coastal regions.

## 1. Introduction

*Margalefidinium polykrikoides* blooms occur in Korean coastal waters in a repetitive manner. This type of bloom has gradually become larger, wider, and more frequent since first appearing in 1995 [1,2]. *M. polykrikoides* blooms with a cell abundance greater than 1000 cells mL^−1^ can have a negative impact on fish or shellfish [3]. Large-scale *M. polykrikoides* blooms first formoffshore and are then generally transported to coastal waters, where they gradually accumulate [4]. Because there are many aquaculture cages found along the south coast of Korea, The occurrence of blooms with a high cell abundance in this area would cause considerable economic damage. Since the outbreak of red tide blooms in 2015, *M. polykrikoides* blooms have not occurred on a large scale (maximum cell abundance >20,000 cell mL^−1^ and duration >40 days) [4] between 2016 and 2020. Nevertheless, *M. polykrikoides* blooms with a cell abundance of over 1000 cells mL^−1^ have occurred on the southern coast of Korea every year from 2016 to 2020. To mitigate the damage caused by red tide blooms, it is important to identify their spatial distribution. Daily red tide reports provided by the National Institute of Fisheries Science (NIFS) include many types of data, including the nature of the causative organisms, cell abundance, and affected and warning areas [5]. NIFS operates a red tide warning system to provide red tide information through field sampling at discrete locations, focusing on *M. polykrikoides*. This system is composed of four levels: red tide emergence notice (>10 cells mL^−1^), red tide notice (>100 cells mL^−1^), red tide alert (>1000 cells mL^−1^), and warning lift. If the red tide observation areas are very wide, field sampling based on discrete locations is limited in terms of manpower, cost, and time. Hence, remote sensing approaches for red tide monitoring can be an effective tool and can overcome the spatial and temporal limitations of field sampling.

Satellite-based red tide detection has been widely studied using ocean color sensors, such as the Sea-viewing Wide Field-of-view Sensor (SeaWiFS), MODerate resolution Imaging Spectroradiometer (MODIS), Medium Resolution Imaging Spectrometer (MERIS), and Geostationary Ocean Color Imager (GOCI) [6,7,8,9,10,11,12,13,14,15,16,17,18,19,20,21,22,23,24]. Red tide blooms are detected by satellite-based products, such as chlorophyll concentration [6,7,8,9] and normalized fluorescence line height [10,11,12,13]. Stump et al. [6] and Tomlinson et al. [7] demonstrated that a chlorophyll anomaly determined by pixel-wise means of a chlorophyll concentration map for 60 days was suitable for detecting *Karenia brevis* blooms. This method was used as an early warning system to forecast *K. brevis* blooms in the eastern Gulf of Mexico from the National Oceanic and Atmospheric Administration. Amin et al. [13] used the fluorescence properties in the red part of the spectrum for red tide detection. Suh et al. [14] detected red tide blooms using the increase in suspended particulate matter (SPM) caused by the occurrence of red tide blooms. In addition, optical properties, such as the absorption coefficient (a), backscattering coefficient (b_bp_), and remote-sensing reflectance (R_rs_) of red tide waters have also been applied to detect red tide blooms [15,16,17,18,19,20,21,22,23,24]. Wynne et al. [15] found that *K. brevis* blooms have the optical properties of low backscattering. Tomlinson et al. [16] used the spectral slope of the normalized water-leaving radiance (_n_L_w_) at approximately 490 nm. The use of this wavelength enables the distinction of *K. brevis* blooms from other algal blooms. Dierssen et al. [23] and Sasaki et al. [24] reported that the peak of R_rs_ shifted to longer wavelengths (570–590 nm) when red tide blooms occur. Owing to their high spectral resolution and signal-to-noise ratio (SNR), ocean color sensors are excellent for detecting red tide patterns, even in conditions mixed with seawater, as well as the presence or absence of red tide blooms. However, ocean color sensors cannot provide accurate red tide information in coastal areas because of their low spatial resolution (300–500 m) and coastal masking caused by the uncertainty of atmospheric correction in coastal areas where turbidity is high [25,26]. Because the gaps between aquaculture cages in coastal areas are fewer than tens of meters, ocean color sensors have limitations with regards to the detection of red tide blooms around these structures.

Terrestrial sensors with a high spatial resolution can overcome the limitations of ocean color sensors. Some studies have employed terrestrial sensors, such as Landsat Enhanced Thematic Mapper Plus (ETM+), Operational Land Imager (OLI), and Sentinel-2 MultiSpectral Instrument (MSI) for red tide detection. Sakuno et al. [27] proposed a simple red tide monitoring method using Sentinel-2 MSI for the sustainable management of small lakes. They used a band combination based on the spectral features of a red tide bloom. Khalili and Hasanlou [28] monitored red tide blooms in coastal areas using the spectral features of Sentinel-2 MSI. Shin et al. [25] attempted spatial and spectral-based image fusion of GOCI and Landsat OLI to improve the accuracy of red tide detection. Landsat OLI was chosen to compensate for the low spatial resolution of GOCI, and as a result, it was possible to obtain reasonable red tide information in both coastal and offshore areas. However, some red tide patterns suffered from severe noise caused by sea currents and ship wakes that appeared mainly in images with a high spatial resolution. In addition, Shin et al. [26] investigated the synergistic effect of GOCI, Sentinel-3 Ocean and Land Colour Instrument (OLCI), Landsat ETM+, OLI, and Sentinel-2 MSI data to identify *M. polykrikoides* blooms in coastal areas. They concluded that the red tide accuracy increased with multi-sensor data rather than with single-sensor data. In addition, they found the red tide patterns near the coastal area on Sentinel-3 OLCI images, but not on Landsat OLI images because terrestrial sensors have a lower spectral resolution and SNR than ocean color sensors. The results of these studies show the limitations of the conventional analyses on remote sensing data, even with a high spatial resolution, to extract red tide information.

To overcome these limitations, machine learning and deep learning approaches have been used. They have been actively applied to earth system sciences to study the atmosphere, land surface, and ocean [29,30,31,32,33,34,35,36,37,38,39]. In particular, machine learning has become a popular approach in geoscientific classification, anomaly detection, and regression. Furthermore, deep learning is used in geoscience to better understand the spatial and temporal structures of data. These data have features that would generally be problematic for traditional machine learning to extract [40]. However, several studies have reported success in applying deep learning to objectively extract spatial features to define and classify various conditions without using either subjective human annotation or a threshold-based method. Recently, some studies have applied machine learning and deep learning for satellite-based red tide detection. Cheng et al. [41] concluded that machine learning techniques such as random forest and support vector machine, outperformed the previous threshold-based methods using MODIS images [8,17]. Lee et al. [42] developed a red tide detection scheme based on a multilayer feed-forward neural network from Landsat OLI images. The detection results showed good agreement with the red tide map from in situ measurements compared to previous red tide indices developed by Lou and Hu [20], Kim et al. [21], and Shanmugam et al. [43]. Shin et al. [44] applied four machine learning approaches to retrieve red-tide cell abundance on the southern coast of Korea from airborne hyperspectral imagery, namely feed-forward neural network, support vector machine, ensemble bagged tree, and Gaussian process regression. The paired data of in situ spectra and red tide cell abundance were used to train and validate the machine learning models. These models produced accurate red tide cell abundance maps. Kim et al. [45] presented a deep convolutional neural network (CNN) model, U-Net, for the automatic pixel-wise detection of red tides in Korean coastal areas from GOCI images. The red tide index maps predicted by the trained U-Net showed considerable matching spatial occurrence tendencies of the three red tide species to ground-truth information. These studies demonstrated the synergistic effect for red tide detection using state-of-the-art machine learning and deep learning approaches, and accumulated satellite images over the years. However, in order to detect red tide blooms in coastal areas, the use of high spatial resolution (<5 m) images is essential. To the best of our knowledge, research on red tide detection using deep learning methods and satellite images with a high spatial resolution has yet to be conducted.

In this study, we developed a deep CNN model, U-Net, for *M. polykrikoides* detection on the southern coast of Korea from PlanetScope imagery with a high spatial resolution of 3 m. We (1) performed visual inspection and spectral analysis of waters containing *M. polykrikoides* using PlanetScope image, (2) determined the optimal threshold of the conventional red tide index (RTI), (3) trained and tested the U-Net model using pairs of PlanetScope images and red tide maps, and (4) evaluated the performance of RTI and the trained U-Net model for *M. polykrikoides* red tide detection.

## 2. Materials and Methods 

### 2.1. Study Area

**Figure 1:**
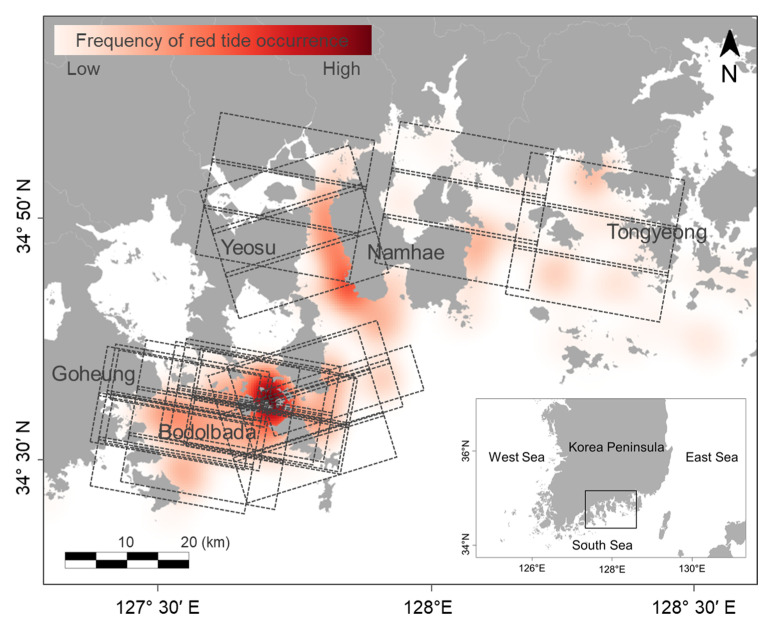
The study area covers the southern coast of Korea, including Goeung, Yeosu, Namhae, and Tongyeong. Black dotted boxes show the acquisition area in PlanetScope imagery between 24 July and 2 August in 2018. The frequency map shows the main regions of red tide occurrence in 2018. These data come from the red tide daily reports provided by NIFS.

The study area covers the southern coast of Korea, including Goheung, Yeosu, Namhae, and Tongyeong, which are composed of complex ria coastline structures (Figure 1). This coast has many islands, and the optical properties of coastal and offshore waters are very different. Offshore water is clear (Case-1 water) because of the Kuroshio Current, but it can also exhibit a complex water characteristic (Case-2 water) caused by colored dissolved organic matter (CDOM) and SPM, which increases toward coastal areas [19,46]. Figure 1 shows the acquisition area of PlanetScope imagery and the frequency map of *M. polykrikoides* blooms in 2018, which has occurred in the last five years on a large scale. On 23 July 2018, a red tide bloom began to occur at an abundance of up to 761 cells mL^−1^ along the coast of Yeosu. Red tide notices were first issued from Goheung to Namhae on 24 July 2018. After the first red tide blooms in the Bodolbada on the 26th, high-density red tide blooms appeared sporadically in the center of the Bodolbada until 31st. On 27 July 2018, the maximum abundance of *M. polykrikoides* was 4500 cells mL^−1^ in Bodolbada. On the 31st, an additional red tide notice was issued for Tongyeong and Geoje. On August 2, the maximum abundances appeared along the coasts of Namhae (1800 cells mL^−1^) and Tongyeong (1200 cells mL^−1^), and then, the blooms slowly disappeared. On August 20, the red tide notice was completely lifted throughout the water. In some waters, high-density red tide blooms above 1000 cells mL^−1^ occurred, but no red tide alert was issued because the blooms did not continue to occur. The black dotted boxes (Figure 1) show the acquisition area of PlanetScope between 24 July and 2 August 2018. Most of the acquired images cover the Bodolbada area between Yeosu and Goheung. Additionally, some images cover the areas of the Tongyeong and Namhae coasts.

### 2.2. Paired Datasets of PlanetScope Imagery and Corresponding Red Tide Maps

In this study, we used PlanetScope imagery to detect the pixel-wise existence of red tides. The PlanetScope was launched in June 2016, and the satellite constellation consists of approximately 130 satellites and can image the entire land surface of the Earth every day [47]. Each PlanetScope satellite is a CubeSat 3U with dimensions of 10 × 10 × 30 cm^3^ and carries a telescope and a frame charge-coupled device (CCD) camera equipped with a Bayer-mask filter. Table 1 shows a constellation overview of the PlanetScope. The orbit altitude is 475 km, and the equator crossing time is between 9:30 am and 11:30 am at local solar time. Images around the Korean Peninsula were obtained at approximately 10:40 am every day. Each image pixel had four spectral bands and a spatial resolution of 3 m. An imagery product covers an area of approximately 24 × 7 km^2^, with some variability depending on the satellite altitude.

Imagery products are available as either individual basic scenes (level 1B), ortho scenes (level 3B), or ortho tile products (level 3A). Nevertheless, we used the orthorectified level 3B surface reflectance (SR) PlanetScope product. Level 3B ortho scenes consist of raw digital number (DN) and SR product suitable for analytic and visual applications. The DN value is converted into Top of Atmosphere (TOA) radiance (*Wm^2^sr^−1^μm^−1^*), then the SR is calculated from the TOA reflectance through an atmospheric correction [47]. We acquired PlanetScope imagery during the red tide event in 2018. Table 2 summarizes the PlanetScope imagery available during the red tide bloom event in the southern coast of Korea in 2018. Except for 28 July, a total of 36 images were collected consecutively from July 24 to August 2. Most images were cloud-free, with the exception of some images.

The ground truths of *M. polykrikoides* red tide maps corresponding to 36 PlanetScope images were obtained from daily red tide reports provided by NIFS [5] (Figure 1). Each pixel value has a discrete binary value that represents the nonexistence and existence of a red tide, respectively. To construct paired red tide map matching with the spatial regions of PlanetScope imagery, the red tide data in polygon format, which were provided by NIFS, were converted into ground-truth maps in raster format. We calculated the areas of red tide blooms on the ground-truth maps corresponding to 36 PlanetScope images. Among the 36 images, three had a red tide area of over 10 km^2^, and the others had areas of less than 5 km^2^. The widest red tide area of 16.81 km^2^ was observed on 29 July 2018.

### 2.3. Red Tide Detection Methods

We performed visual inspection and spectral analysis using the 3B SR PlanetScope images. The optical spectra of various types were extracted, and then, we applied the conventional RTI to PlanetScope images. RTI was developed by Oh et al. [48] for the detection of red tide blooms from unmanned aerial vehicle (UAV) images. It utilizes the optical features of the red tide waters. The water-leaving radiance of clear seawater and red tide water are significantly different in the blue and green wavelength bands [49]. However, the turbidity of seawater makes it difficult to differentiate the spectral characteristics of red tide waters from highly turbid seawater. Because the waters on the southern coast of Korea have complex optical properties, the reflectance of red tide waters is largely different from that of highly turbid waters in the red wavelength band [19]. The RTI was calculated using the reflectance in the red, green, and blue wavelength bands as follows [48]:

(1) $$\mathrm{Red}\;\mathrm{Tide}\;\mathrm{Index}=\frac{\left(R_{green\;band}\;-\;R_{blue\;band}\right)}{\left(R_{green\;band}+R_{blue\;band}\right)}+\frac{\left(R_{red\;band}\;-\;R_{blue\;band}\right)}{\left(R_{red\;band}+R_{blue\;band}\right)}$$

Then, a threshold was applied to differentiate red tide waters from seawater. Originally, it was flagged as red tide water when the RTI value was > 0.2. However, the threshold is dependent on sensors, and even images acquired by the same sensor can have different optimal thresholds. We compared the statistics of the RTI distributions of non-red tide and red tide pixels, and then determined the optimal thresholds to differentiate the two RTI distributions of each image.

**Figure 2:**
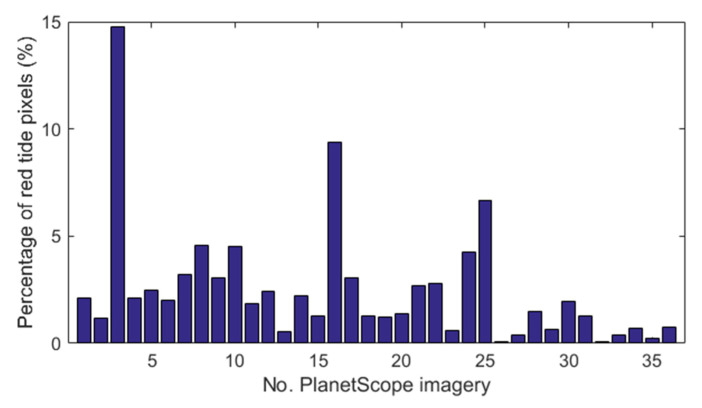
The percentage of red tide pixels in each PlanetScope image.

Next, a deep CNN, U-Net, was trained with PlanetScope imagery and the corresponding ground-truth red tide maps were used as both input and output. A total of 36 image pairs were used to develop the U-Net model to learn the spectral features of red tides from PlanetScope images. The deep convolutional layers in a CNN extract spectral features related to red tide waters from multi-band images. We adopted the U-Net CNN architecture for red tide detection through deep learning pixel-wise spectral features from the PlanetScope images. U-Net is a “U”-shaped CNN with encoder and decoder structures, and it is widely applied to pixel-wise classification or object segmentation [50]. U-Net can be trained end-to-end from very few images and has been found to outperform the previous best method. Moreover, in this study, the network was fast and had a reasonable training time. Figure 2 shows the percentage of red tide pixels in each image from 24 July to 2 August 2018. The portion of red tide pixels among the 36 images was 2.56%, and non-red tide pixels significantly outnumbered red tide pixels. In addition, the size of the entire image (for example, 4620 × 9004 pixels on 29 July 2018) was too large to pass through the U-Net model. To efficiently train the U-Net model, we split the PlanetScope image and ground-truth map into spatially non-overlapped patches of 32 × 32 pixels. Then, we used only patches with more than one red tide pixel as the training dataset. As a result, we generated 24,958 valid patch pairs between the PlanetScope images and corresponding ground-truth maps. In 24,958 matched pairs, the percentages of red tide and non-red tide pixels were 55% and 45%, respectively. In order to identify the effect of randomness on patch selection and the proportion of red tide pixels, we constructed four different training and test datasets: (1) U-Net #1 (the ratio of randomly selected non-red tide pixels: red tide pixels = 0.45:0.55); (2) U-Net #2 (non-randomly selected non-red tide: red tide = 0.45:0.55); (3) U-Net #3 (randomly non-red tide: red tide = 0.66:0.34); and (4) U-Net #4 (non-randomly non-red tide: red tide = 0.66:0.34). Datasets of U-Net #1 and #2 only contained patches with at least more than one red tide pixel; on the other hand, U-Net #3 and #4 contained additional patches consisting of non-red tide pixels. To validate the trained U-Net model, test datasets were constructed in approximately 25% of the training datasets, maintaining a similar ratio of red tide and non-red tide pixels as that of the training datasets. 

**Figure 3:**
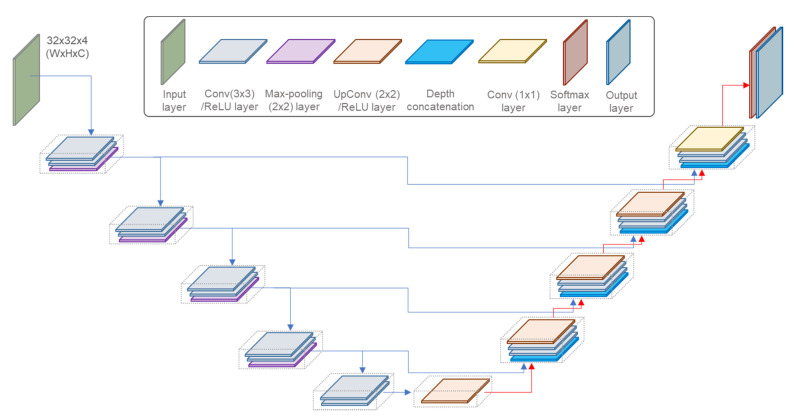
U-Net architecture for red tide detection from PlanetScope image.

U-Net was trained to classify four spectral bands of each pixel in 32 × 32 PlanetScope patches to two labels of zero and one, corresponding to non-red tide and red tide, respectively. Here, U-Net was constructed with five convolutional layers and five deconvolutional layers to extract spectral features related to red tide waters from the four wavelength bands of the PlanetScope images. As shown in Figure 3, the encoder block is down from the top to the bottom layers on the left to extract from coarse to fine features. The extracted features pass through the decoder block from the bottom to the upper layers on the right side. The kernel size and number of convolutional layers at the encoder and decoder blocks are summarized in Table 3. We added a dropout layer in the end of the encoder block. The softmax and output layers determine whether each pixel is related to a red tide. The loss function (L) consist of a cross-entropy (*L_CE_*) and L2 regularization (*L*_2_) functions with a hyper-parameter (λ), as defined in Equations (1) and (2) [51]. In Equation (2), *t_ij_* and *y_ij_* are the indicators and the softmax output for the *i*-th sample and the *j*-th label, respectively. *N* and *K* are the number of samples and labels, respectively.

(2) $$L\left(\theta \right)=L_{CE}\left(\theta \right)+\lambda L_{2}\left(w\right)$$

(3) $$L_{CE}\left(\theta \right)=\sum_{i=1}^{N}\sum_{j=1}^{k}t_{ij}\ln\left(y_{ij}\right)\;and\;L_{2}\left(w\right)=\frac{1}{2}w^{T}w$$

To train the U-Net from the given datasets, a stochastic gradient descent with momentum (SGDM) optimizer [52] was used with different training options: 10, 25, and 50 mini-batches, and 10, 25, and 50 epochs. All batches were normalized. Datasets were shuffled at every epoch during training, and the learning rate and hyperparameter of L2 regularization were set to 0.05 and 0.0001, respectively.

### 2.4. Performance Assessment

To evaluate the performance for red tide detection, we performed qualitative and quantitative assessments of the RTI and U-Net models trained with four different datasets. For qualitative assessment, we visually compared the ground-truth red tide map and the red tide maps generated through RTI and four U-Net models from a PlanetScope image taken on 29 July 2018. We quantitatively assessed the performance using a confusion matrix to evaluate the accuracy of red tide detection [53]. In the case of the four U-Net models, we utilized patches and 36 images. For RTI, the evaluation was performed by applying predetermined thresholds to 36 images. Lastly, the performance of the RTI and U-net models on 36 images was compared. In Table 4, the *mr* and *nmr* symbols indicate the red tide and non-red tide pixels in the ground-truth red tide maps, respectively. *MR* and *nMR* indicate red tide and non-red tide pixels in the predicted red tide maps, respectively.

Four figure-of-merits (FOMs), namely accuracy, sensitivity, precision, and F-measure, were calculated from the confusion matrix. The accuracy was calculated as [(1) + (4)]/[(1) + (2) + (3) + (4)] for all pixels in the red tide map. It is the most intuitive performance evaluation indicator; however, if data are biased, the accuracy may be limited in interpreting the performance of the model. Additionally, the sensitivity ((1)/[(1) + (2)]) and precision ((1)/[(1) + (3)]) were evaluated using only red tide pixels in the ground-truth and predicted red tide maps. The F-measure, which is the harmonic mean of precision and sensitivity, was evaluated as follows [53]:

(4) $$F-\mathrm{measure}=\frac{2\times \;\mathrm{Precision}\;\times \mathrm{Sensitivity}}{\mathrm{Precision}\;+\mathrm{Sensitivity}}$$

## 3. Results

### 3.1. Visual Inspection and Spectral Analysis

**Figure 4:**
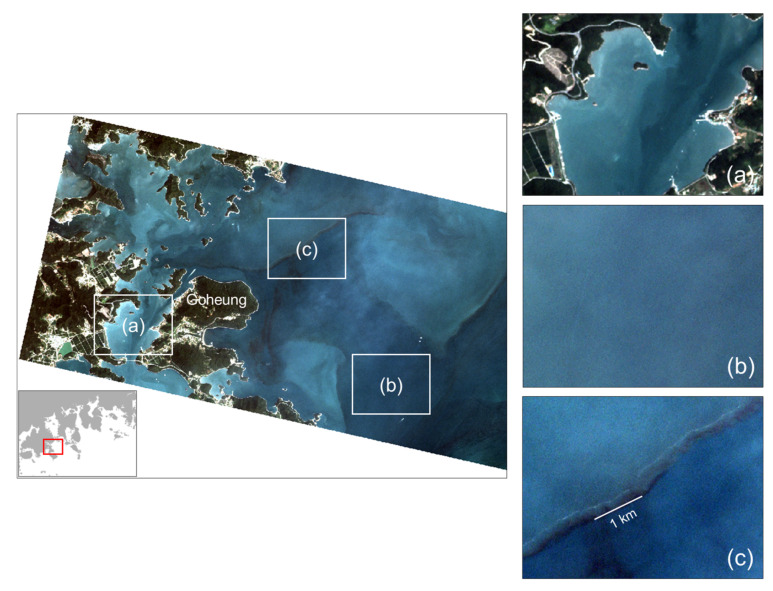
3B surface reflectance (SR) PlanetScope true-color composite image (R: 630 nm; G: 545 nm, B: 485 nm) acquired near the coast of Goheung on 29 July 2018 at 01:42 GMT. Selected regions, (**a**–**c**) in the left image are enlarged on the right panels. Regions (**a**–**c**) represent turbid, the surrounding, and red tide waters, respectively. Region (**c**) shows a high-density *M. polykrikoides* strip in brown appearing along the coast of Goheung (2500 cells mL^−1^).

We investigated three water types: turbid, red tide, and the surrounding waters from a PlanetScope true-color composite image on 29 July 2018 at 01:42 GMT for visual inspection (Figure 4). The regions were selected based on ground-truth red tide information. Figure 4a shows the turbid waters between the islands. This region is an area where no red tides occur in the ground-truth red tide information. The turbid waters near the coast were brighter than the surrounding seawater, indicating a high SPM. *M. polykrikoides* red tide patterns are generally brown or reddish in true color composite images. In the case of the surrounding waters in Figure 4b, no red tide bloom was found in the daily report. As shown in Figure 4c, the clear pattern of *M. polykrikoides* in brown was distinguishable from the surrounding seawater through simple visual analysis. A red tide notice was issued from Goheung to Yeosu according to a daily red tide report on the same day. *M. polykrikoides* blooms appeared continuously on the coast of Goheung and Yeosu (30-920 cells mL^−1^). In particular, a daily report indicated that high-density red tide strips appeared along the coast of Goheung (2500 cells mL^−1^). 

**Figure 5:**
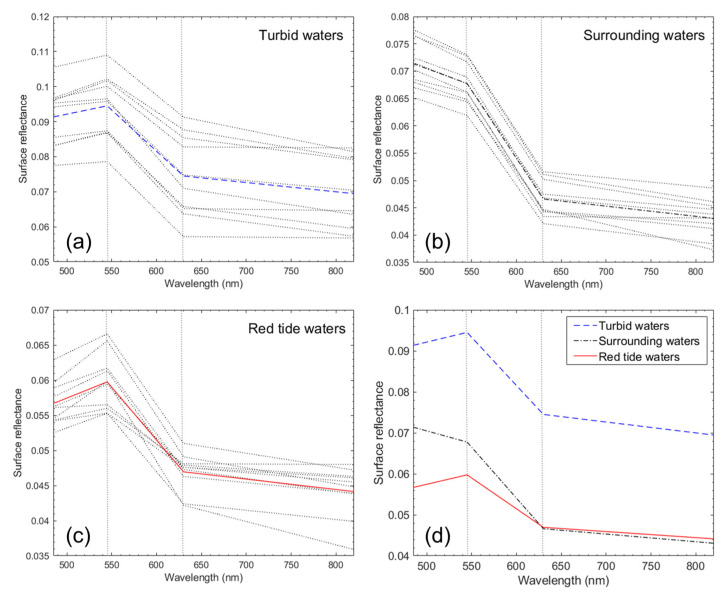
Spectra of (**a**) turbid waters, (**b**) the surrounding water, (**c**) red tide waters, and (**d**) the average spectra of three water types in the coast of Goheung from 3B SR PlanetScope image on 29 July 2018 at 01:42 GMT. For spectra extraction of the three water types, pixels were randomly selected from the regions in Figure 3a–c. Blue dashed, black dash-dot, and red lines indicate the average over the extracted spectra in (**a**–**c**), respectively.

Figure 5 shows spectra of turbid, the surrounding, and red tide waters extracted from 3B SR PlanetScope image. All spectra were acquired near the coast of Goheung on 29 July 2018 at 01:42 GMT. Pixels were randomly selected from Figure 4a–c for spectra comparison of the three water types. For turbid water, in Figure 5a, a bright pixel near the coast was chosen. The greatest SR spectral values were observed in the green band of 545 nm and tended to decrease at shorter and longer wavelength bands. These properties appeared in complex waters (Case-2 water), which had high levels of SPM and CDOM. As result of spectral analysis in Figure 5b,c, spectra in the waters containing red tide represent the typical shape of *M. polykrikoides* patches compared to the surrounding waters. This is consistent with spectra of *M. polykrikoides*, shown in Landsat images with wavelength bands similar to PlanetScope [23]. However, although the magnitude of the SR value in turbid waters was greater than that of the SR value in red tide waters, it is quite difficult to distinguish the two spectra based on spectra shape because the two spectra are very similar in Figure 5a,c.

### 3.2. Threshold Determination of RTI

**Figure 6:**
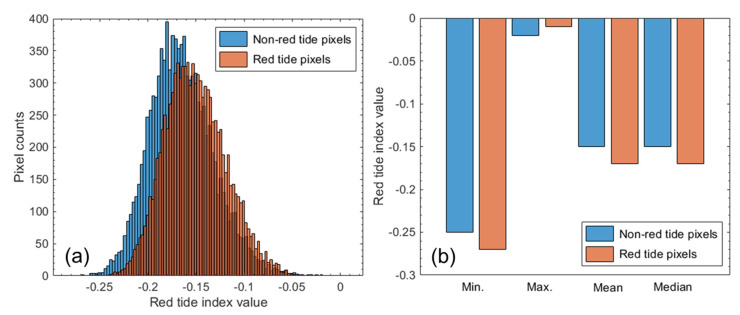
(**a**) Histograms of red tide index (RTI) values in relation to 10,000 non-red tide pixels and 10,000 red tide pixels in a PlanetScope image acquired on 29 July 2018 01:42 GMT near the coast of Goheung. (**b**) The statistics calculated from RTI distribution of randomly-selected pixels of each group. Min. and Max. are minimum and maximum values, respectively.

**Figure 7:**
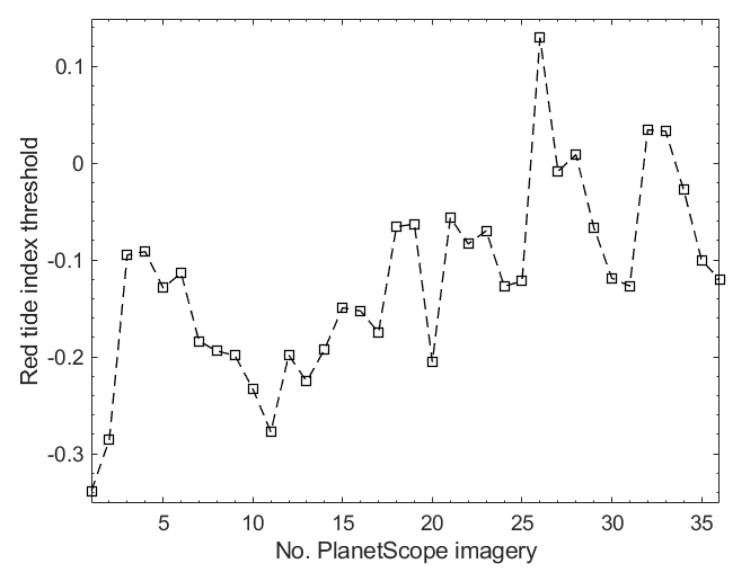
RTI thresholds of 36 PlanetScope images. The threshold was determined by the median RTI value of the red tide pixel group in each image.

Figure 6 shows the histograms and their statistical properties of non-red tide and red tide pixels determined by RTI calculated from the wavelength bands of a PlanetScope image acquired on 29 July 2018 at 01:42 GMT, where 10,000 pixels were selected randomly from the non-red tide and red tide pixel groups. As shown in Figure 6a, the RTI histograms of both groups showed a symmetrical bell-shaped distribution. Figure 6b shows the RTI values for both groups. The RTIs in both groups were distributed in a similar range. The minimum RTI of the non-red tide group was larger than that of the red tide group, whereas the opposite was true for the maximum RTI. Both groups showed similar mean and median RTI values. From the overlapped histograms and their similar statistics for both groups, we concluded that RTI was not sufficient to distinguish between red tide and non-red tide pixels via a threshold. Figure 7 shows the RTI thresholds determined by the median RTI values of the red tide group for 36 PlanetScope images. We recognized the red tide areas when the RTI value was above the threshold determined for each image. 

### 3.3. U-Net Model for Deep Red Tide Learning

To determine the optimal size of a mini-batch, we tested three mini-batches of 10, 25, and 50 using the training dataset of U-Net #1. If the mini-batch size was 50, the number of iterations per epoch was 399. We confirmed that the training loss of the mini-batch size of 50 decreased faster than that of the mini-batch size of 10. Among the three epochs of 10, 25, and 50, epoch 50 showed the highest FOMs. Thus, we trained the U-Net models with four different training and test datasets using a mini-batch size of 50 and epoch 50. Table 5 shows the performance of the four U-Net models using patches for the training and test datasets and 36 PlanetScope images. First, we calculated the FOMs using the patches. As a result, the FOMs of the training and test datasets are similar. In the case of the test dataset, while U-Net #4 showed the highest accuracy and U-Net #2 had the highest sensitivity level. Except for U-Net #3, the other models showed similar FOM levels. In the case of 36 images, U-Net #3 showed the highest accuracy and precision, whereas U-Net #2 showed the highest sensitivity. In terms of the F-measure, which combine precision and sensitivity, U-Net #4 provided the highest level. Compared to U-Net #1, trained by datasets chosen randomly, the sensitivity of U-Net #2 trained by datasets chosen non-randomly increased by 10.39%, while other FOMs decreased (accuracy by 5.64%, precision by 28.10%, and F-measure by 25.26%). Compared to U-Net #3, the accuracy and precision of U-Net #4 decreased by 6.14% and 12.11%, respectively, but the sensitivity and F-measure levels increased by 53.69% and 9.44%, respectively. In the case of U-Net #1 and U-Net #3, trained with different ratios of non-red tide and red-tide pixels chosen randomly, the higher the proportion of non-red tide pixels, the higher the sensitivity by 43.46%; however, the higher the other FOMs (accuracy by 14.99%, precision by 81.71%, and F-measure by 25.04%, respectively). In the case of U-Net #2 and U-Net #4 trained with non-randomly chosen data, the FOMs showed similar trends to the FOM comparison of U-Net #1 and U-Net #3. In summary, the lower the proportion of red tide pixels, the higher the F-measure level, up to an 83% increase. Comparing the four FOMs in Table 5, we chose U-Net #4, which provided the best F-measure. 

### 3.4. Performance Comparison of RTI and U-Net

**Figure 8:**
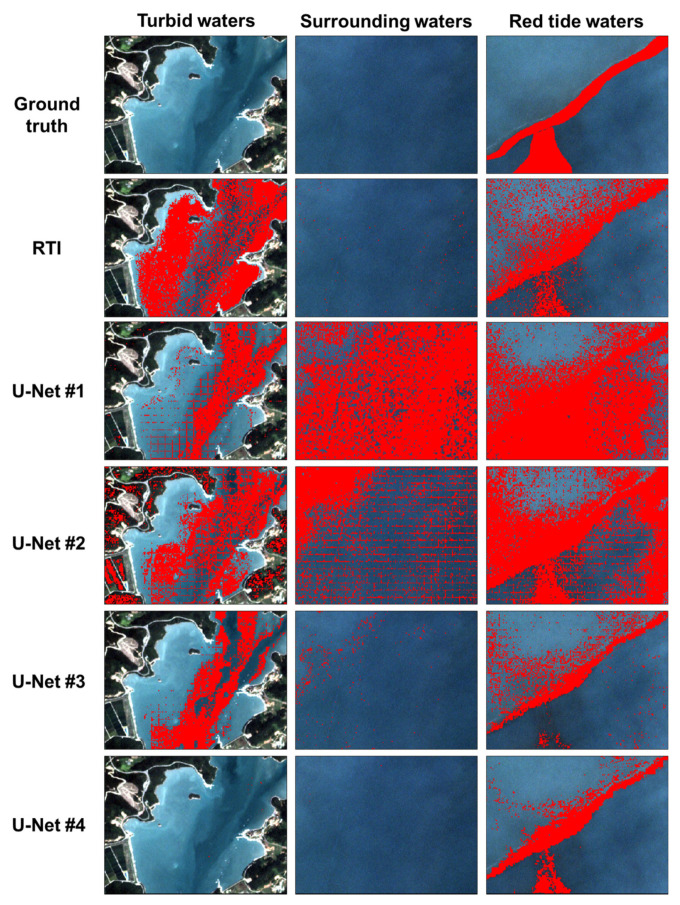
Ground-truth and red tide maps generated by RTI and four U-Net models in the three water types of turbid, surrounding, and red tide waters from a PlanetScope image acquired on 29 July 2018 at 01:42 GMT near the coast of Goheung. Red tide pixel was marked in red.

Figure 8 shows ground-truth and red tide maps generated through RTI and four U-Net models from a PlanetScope image around the coast of Goheung on 29 July 2018, at 01:42 GMT. Referring to the ground truths, the red tide pattern from RTI appeared relatively appropriate. However, RTI tended to identify the turbid waters as the red tide waters. In the red tide map based on U-Net #1 and U-Net #2, which were trained using only red tide patches, red tide areas were overestimated in all water types. In particular, the edge pixels of each patch tended to be identified as red tide pixels. Even some land areas were recognized as red tide pixels. On the other hand, red tide maps based on U-Net #3 and U-Net #4, which were trained with the mixed red tide and non-red tide patches, showed reasonable red tide patterns compared to the other U-Net models. However, in the case of U-Net #3, the edge pixels of patches and the turbid waters were still recognized as red tide pixels. U-Net #4 trained with non-randomly selected non-red tide and red-tide patches showed the most reasonable red tide patterns in all water areas.

**Figure 9:**
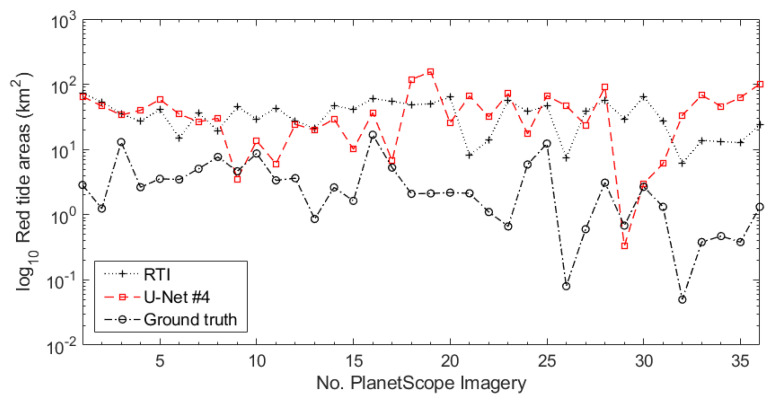
Red tide areas extracted from RTI, U-Net #4, and ground-truth maps of 36 PlanetScope images.

We compared the quantitative performances of RTI and U-Net #4 for the 36 PlanetScope images in Table 6. We evaluated FOMs for three different areas of red tide occurrence: low extent (LE), middle extent (ME), and high extent (HE) groups. LE indicates red tide areas <5 km^2^, ME refers to areas of 5–10 km^2^, and HE refers to an area of more than 10 km^2^. For the performance of the RTI and U-Net #4, the accuracy was similar in each group. However, compared with the RTI, the FOMs of U-Net #4 were improved. The sensitivity of U-Net #4 in the LE and HE groups increased by 22% and 32%, respectively. The precision of U-Net #4 in LE, ME, and HE increased by 33%, 78%, and 40%, respectively. The F-measure of U-Net #4 in the ME and HE groups increased by 57% and 35%, respectively. Figure 9 shows the red tide areas extracted from the RTI, U-Net #4, and ground-truth maps of 36 PlanetScope images. The red tide areas extracted from the RTI and U-Net #4 were wider than those in the ground-truth areas. We compared the difference between the ground truth areas and red tide areas extracted using the RTI and U-Net #4. As a result, red tide maps extracted by RTI did not differ by less than 5 km^2^ from the ground-truth area, while red tide maps derived from U-Net #4 differed by less than 5 km^2^, accounting for 19% of the 36 images. The differences in the red tide areas between the RTI and U-Net #4 with the ground truth were similar in the LE and HE groups. However, for the ML group, U-net #4 (10.31 km^2^) differed significantly less than in RTI (24.23 km^2^).

## 4. Discussion

Despite the high spatial resolution of the PlanetScope images, it was very difficult to distinguish red tide pixels from non-red tide pixels due to low spectral resolutions and SNR. To overcome the limitations of PlanetScope, we used the U-Net model to better exploit the spatial and spectral features of the images. In this study, the U-Net model provided more reasonable red tide information from high spatial resolution imagery in coastal areas than the traditional threshold-based method. However, several factors affect the performance of predicting red tide maps using RTI and U-Net models.

### 4.1. PlanetScope Product

PlanetScope imagery has been available since 2016, but it was only in 2018 that cloud-free images along the southern coast of Korea could be obtained continuously during a red tide event. Therefore, we conducted a study in 2018. The PlanetScope satellite constellation consists of groups of individual satellites. In this study, we used PlanetScope images from the three satellites. Of the 36 images, the number of satellite IDs 10xx, 0fxx, and 0exx were 20, 12, and 4, respectively. In some cases, two satellite ID images were acquired with a slight time difference on the same date. The three satellites exhibit different relative spectral responses. Satellite IDs 0fxx and 0exx showed similar spectral response curves, but the satellite ID 10xx most utilized in this study showed very different curves from those of different satellite IDs. To determine the effect of this difference on the actual spectrum, we performed an analysis of the red tide spectrum at the same location obtained from different satellite images of the same date. The spectral shapes did not show significant differences, but minor variations in magnitude were observed. These factors could affect the spectral analysis of red tide blooms and affect the performance of our U-Net model because the deep convolutional layers in the U-Net architecture learn by extracting spectral features related to red tide waters from multi-band SR PlanetScope images. Compared to ocean sensors, terrestrial sensors such as PlanetScope have a low spectral resolution and a small band number. The algorithms for red tide detection typically use blue and green bands. This is because these wavelengths allow us to distinguish not only the surrounding waters but also the red tide waters. Therefore, the absence of these wavebands can be a major cause of the difficulty in detecting red tide blooms. In addition, because terrestrial sensors have lower SNRs than ocean color sensors, the utilization of terrestrial sensors for red tide detection leads to poor performance for red tide pixels mixed with seawater or turbid waters. The Planet SR product used in this study was corrected by combining standard atmospheric models with the use of water vapor, ozone, and aerosol data derived from MODIS [47]. This provides reliable and consistent SR scenes over PlanetScope’s varied constellation of satellites. However, some limitations remain, including the effects of haze and thin cirrus clouds in each image. In fact, of the majority of the 36 images were cloud-free, while four images had cloud cover (20–50%). However, we confirmed that the degree of cloud cover did not significantly affect the performance of the U-Net model. This area also has complex optical properties that may affect the accuracy of atmospheric correction. Despite these limitations, the U-Net model showed reliable results for red tide detection. Therefore, we believe that the U-Net model will also show reliable results for other red tide events, using 36 images with varying atmospheric conditions and sea environments, even if only for nine consecutive days.

### 4.2. Ground Truth Red Tide Map

As shown in Figure 9, the red tide areas extracted from the RTI and U-Net models may differ from the daily red tide reports provided by the NIFS. The daily red tide report is generated by summarizing fishermen’s reports and a daily field survey of red tide blooms [5]. The extent of red tide extent from daily reports can be regarded as the extent of red tide blooms that occurred throughout the day. Because PlanetScope images were acquired at approximately 10:40 GMT+9, the red tide areas extracted in this study may not represent the red tide distribution for the whole day. In addition, daily red tide reports provide information on areas where red tides occur frequently. Therefore, the information in the report is not indicative of red tide information that occurred all over the waters. The actual red tide areas can be recognized as non-red tide areas in the ground-truth data. Hence, these samples were used to train the U-Net model. The difference in the data format between the ground-truth red tide map and PlanetScope imagery may affect the performance of the RTI and U-Net models. While NIFS provides red tide spatial information in polygon format, PlanetScope image are given in raster format; thus, we converted the NIFS datasets in polygon format to raster format. Depending on the spatial resolution in raster format, the areas of red tide produced after conversion can fluctuate, causing uncertainty with regards to the extent of the red tide. Above all, *M. polykrikoides* is characterized by vertical migration [2,54,55]. *M. polykrikoides* is known to move on the sea surface in the morning (beginning at approximately 8:00 GMT+09), with the surface cell density peaking around 16:00 GMT+09, and decreasing thereafter. In this study, PlanetScope images were acquired at approximately 10:00 or 11:00 GMT+09. Hence, the acquisition time of the PlanetScope images did not correspond to the peak time of the red tide bloom. Therefore, this could explain the lower performance of the RTI and U-Net models.

In addition to *M. polykrikoides* blooms, a variety of harmful and non-harmful blooms occur frequently near the coast. The optical properties of these red tide species are very similar, and it is not easy to distinguish *M. polykrikoides* from other red tide species. In order to distinguish these red tide species, it is essential to collect the ground truth for each red tide species. However, NIFS provides red tide information focused on *M. polykrikoides* blooms because the red tide monitoring system was established after a massive fish-kill by *M. polykrikoides* blooms in 1995. For this reason, information on the occurrence of other red tide species is difficult to obtain without a field survey. In fact, NIFS provided only information on *M. polykrikoides* blooms between 24 July and 2 August 2018, when the PlanetScope images were acquired. If blooms were caused by red tide species not provided by NIFS during this period, this would cause a difference between the extracted areas and ground-truth areas. Although the red tide events were detected from PlanetScope images, the events were not monitored by NIFS. Therefore, it needs to be considered as a potential red tide area.

### 4.3. Red Tide Extent 

The red tide extent associated with red tide intensity can affect the performance of predicting a red tide map. Both the RTI and U-Net models utilize the spectral characteristics of red tide water. If the real intensity of red tide blooms is weak, the unique spectral characteristics of the red tide are not well represented in the satellite images. Red tide attention was issued during the PlanetScope image acquisition period. However, no red tide blooms with high densities were found in any of the images. Red tide intensity generally showed a positive relationship with the extent of red tides. Red tide attention is usually issued by the criteria of red tide cell density (>10 cells mL^−1^), and the criteria have a scale of red tide extent. Red tide blooms occur over a radius of 2–5 km (12–79 km^2^). These criteria imply that the red tide intensity and the extent of the red tide are related. The red tide extent for the individual images was calculated based on the ground-truth red tide map. Among the 36 images, only three showed a red tide extent of 10 km^2^ or more. Other images showed a red tide extent of less than 5 km^2^. Some images even showed a red tide extent of less than 1 km^2^. In fact, the relationship between the extent of red tides and the F-measure level for each image showed a positive correlation. In the case of RTI and U-Net #4, the R^2^ values between red tide extent and F-measure are 0.67 and 0.71, respectively. As shown in Table 6, the F-measure of RTI and U-Net #4 in the LE group was low, while that in the HE group was at a reasonable level. This means that the wider the red tide extent in the image, the higher the performance of the predicted red tide map.

### 4.4. Patch Selection

In the case of the U-Net model, randomness in patch selection and the proportion of non-red tide pixels affect the performance of the predicted red tide map. For non-random sampling, we selected a continuous patch around the region where the red tide occurred on each image. In the four U-Net models, the red tide area of the non-random case was less overestimated than that of the random case. When we constructed training datasets with spatially-adjacent patches, it resulted in a higher performance than when patches were randomly selected. We confirmed that non-random sampling can guarantee to the selection of informative red tide patches for good training. In relation to the ratio of non-red tide and red tide pixels, we confirmed that, when using only red tide patches, the edge and non-red tide pixels were recognized as red tide pixels. This is most likely due to the lack of learned knowledge of non-red tide patches, and because the U-Net model is unable to recognize non-red tide pixels well. As a result, the F-measure level increased as the proportion of non-red tide pixels increased. We confirmed the importance of constructing training datasets for the U-net model to reduce the misdetection rate of red tide pixels. These results show that the U-Net model exploits both spatial and spectral features for red tide detection. Furthermore, the adjustment of patch selection allows for overcoming edge misdetection and potential errors resulting from the limitations of the terrestrial sensor.

## 5. Conclusions

In this study, a U-Net deep learning model was developed for *M. polykrikoides* red tide detection on the southern coast of Korea from PlanetScope imagery with a high spatial resolution of 3 m. The major results are as follows: 

(1) We identified a distinct *M. polykrikoides* red tide pattern that distinguishes it from the surrounding waters using the PlanetScope image. Spectral analysis of the PlanetScope image revealed that the spectra in red tide waters showed a typical pattern of *M. polykrikoides* red tide bloom. However, the spectral pattern in turbid waters is similar to that in red tide waters. Therefore, we confirmed that it is difficult to distinguish the spectra of red tide and turbid water using spectral shapes.

(2) To find the optimal RTI thresholds for PlanetScope images, we performed a statistical analysis of red tide pixel and non-red tide pixel groups. From the overlapped histograms and their similar statistics for both groups, we confirmed that RTI may be insufficient to distinguish between red tide and non-red tide pixels via a threshold. We determined the median RTI of the red tide group for the PlanetScope image as a threshold.

(3) We trained and tested four U-Net models to identify the effects of randomness on patch selection and the proportion of non-red tide pixels. As a result, the U-net #4 model, which was selected non-randomly even including non-red tide patches, showed the highest performance in qualitative and quantitative assessments compared to RTI and other U-net models. The predicted red tide map derived from the U-net #4 model showed the most reasonable red tide patterns in all water areas.

Our U-Net model was trained and tested using the spectra of optically complex waters along the southern coast of Korea. Compared to RTI, which utilizes only the spectral characteristics of red tide waters, we confirmed that the U-Net model provides a reasonable *M. polykrikoides* bloom distribution. We believe that these results will not only confirm the utility of U-Net for red tide detection but also provide a foundation for recognizing the potential of red tide utilization using terrestrial sensors with a high spatial resolution.

## Figures and Tables

**Table 1:** Constellation overview of PlanetScope.

Mission Characteristics	Sun-Synchronous Orbit
Orbit altitude	475 km
Equator crossing time	9:30–11:30 am (local solar time)
Spectral bands	Blue: 455–515 nm Green: 500–590 nm Red: 590–670 nm NIR: 780–860 nm
Spatial resolution	3 m
Product size	24 × 7 km^2^

**Table 2:** PlanetScope imagery available during *M. polykrikoides* bloom event in the southern coast of Korea, 2018.

Date	July 2018								August 2018	
	24	25	26	27	28	29	30	31	1	2
Number of available images	4	2	2	3	0	6	2	4	8	5
Total	36									

**Table 3:** Summary of encoder and decoder blocks of U-Net.

Left Side of U-Net		Right Side of U-Net	
Blocks	Layers per Block	Blocks	Layers per Block
Input	Input layer (32 × 32 × 4)	Output	Ouput layer Softmax layer
Encoder-1 to 4	Conv (3 × 3)/ReLU Conv (3 × 3)/ReLU Max pooling	Decoder-5	Conv (1 × 1) Conv (3 × 3)/ReLU Conv (3 × 3)/ReLU Depth concatenation
		Decoder-2 to 4	UpConv (2 × 2)/ReLU Conv (3 × 3)/ReLU Conv (3 × 3)/ReLU Depth concatenation
Encoder-5	Conv (3 × 3)/ReLU Conv (3 × 3)/ReLU	Decoder-1	UpConv (2 × 2)/ReLU

**Table 4:** A confusion matrix for evaluation of red tide detection accuracy.

		Red Tide Index in Ground Truth	
		True (*mr*)	False (*nmr*)
Red tide index in the predicted	True (MR)	(1) True positive	(3) False positive
	False (nMR)	(2) False negative	(4) True negative

**Table 5:** Quantitative performance of U-Net models trained (50 mini-batches, 50 epochs) with four different training and test datasets. Four datasets were chosen randomly or non-randomly with different ratios of non-red tide and red tide pixels. Four FOMs of the models were evaluated using patches of test datasets and 36 PlanetScope images.

	Random Sampling	Pixel Ratio		Patches of Test Datasets				36 PlanetScope Images			
		Non-Red Tide Pixel	Red Tide Pixel	Accu.	Sens.	Prec.	F-Measure	Accu.	Sens.	Prec.	F-Measure
U-Net #1	Yes	0.45	0.55	0.61	0.73	0.62	0.67	0.82	0.69	0.05	0.08
U-Net #2	No	0.45	0.55	0.60	0.74	0.61	0.67	0.77	0.76	0.03	0.06
U-Net #3	Yes	0.66	0.34	0.65	0.37	0.48	0.42	0.94	0.39	0.09	0.10
U-Net #4	No	0.66	0.34	0.71	0.61	0.61	0.61	0.88	0.60	0.07	0.11

Accu.: Accuracy; Sens.: Sensitivity; Prec.: Precision.

**Table 6:** Performance evaluation of RTI and U-Net #4 model using 36 PlanetScope images. FOMs were evaluated according to the red tide extent.

	Accuracy			Sensitivity			Precision			F-Measure		
	LE	ME	HE	LE	ME	HE	LE	ME	HE	LE	ME	HE
RTI	0.90	0.75	0.87	0.50	0.42	0.50	0.03	0.09	0.15	0.06	0.14	0.23
U-Net#4	0.87	0.79	0.89	0.61	0.42	0.66	0.04	0.16	0.21	0.06	0.22	0.31

LE: low extent; ME: middle extent; HE: high extent.

## References

[1] Lee C.K., Park T.G., Park Y.T., Lim W.A. (2013). Monitoring and trends in harmful algal blooms and red tides in Korean coastal waters, with emphasis on *Cochlodinium polykrikoides*. Harmful Algae.

[2] National Institute of Fisheries Science (NIFS) (2015). Harmful Algal Blooms in Korean Coastal Waters.

[3] Gobler C., Gobler C.J., Anderson O.R., Berry D.L., Burson A., Koch F., Rodgers B., Koza-Moore L., Goleski J., Allam B. (2008). Characterization, dynamics, and ecological impacts of harmful *Cochlodinium polykrikoides* blooms on eastern Long Island, NY, USA. Harmful Algae.

[4] Jeong H.J., Lim A.S., Lee K., Lee M.J., Seong K.A., Kang N.S., Jang S.H., Lee K.H., Lee S.Y., Kim M.O. (2017). Ichthyotoxic *Cochlodinium polykrikoides* red tides offshore in the South Sea, Korea in 2014: I. Temporal variations in three-dimensional distributions of red-tide organisms and environmental factors. Algae.

[5] Forecast·Breaking News of the National Institute of Fisheries Science (NIFS). http://www.nifs.go.kr/redtideInfo.

[6] Tester P.A., Stumpf R.P., Steidinger K.A. (1998). Ocean color imagery: What is the minimum detection level for *Gymnodinium* breve blooms. Harmful Algae.

[7] Stumpf R.P., Culver M.E., Tester P.A., Tomlinson M., Kirkpatrick G.J., Pederson B.A., Truby E., Ransibrahmanakul V., Soracco M. (2003). Monitoring *Karenia brevis* blooms in the Gulf of Mexico using satellite ocean color imagery and other data. Harmful Algae.

[8] Tomlinson M.C., Stumpf R.P., Ransibrahmanakul V., Truby E.W., Kirkpatrick G.J., Pederson B.A., Gabriel A.V., Heil C.A. (2004). Evaluation of the use of SeaWiFS imagery for detecting *Karenia brevis* harmful algal blooms in the eastern Gulf of Mexico. Remote Sens. Environ..

[9] Ahn Y.H., Shanmugam P. (2006). Detecting the red tide algal blooms from satellite ocean color observations in optically complex Northeast-Asia Coastal waters. Remote Sens. Environ..

[10] Hu C., Muller-Karger F.E., Taylor C.J., Carder K.L., Kelble C., Johns E., Heil C.A. (2005). Red tide detection and tracing using MODIS fluorescence data: A regional example in SW Florida coastal waters. Remote Sens. Environ..

[11] Moradi M., Kabiri K. (2012). Red tide detection in the Strait of Hormuz (east of the Persian Gulf) using MODIS fluorescence data. Int. J. Remote Sens..

[12] Zhao J., Temimi M., Ghedira H. (2015). Characterization of harmful algal blooms (HABs) in the Arabian Gulf and the Sea of Oman using MERIS fluorescence data. ISPRS J. Photogramm. Remote Sens..

[13] Amin R., Zhou J., Gilerson A., Gross B., Moshary F., Ahmed S. (2009). Novel optical techniques for detecting and classifying toxic dinoflagellate *Karenia brevis* blooms using satellite imagery. Opt. Express.

[14] Suh Y.S., Jang L.H., Lee N.K., Ishizaka J. (2004). Feasibility of red tide detection around Korean waters using satellite remote sensing. Fisher Aqua. Sci..

[15] Wynne T.T., Stumpf R.P., Tomlinson M.C., Warner R.A., Tester P.A., Dyble J., Fahnenstiel G.L. (2008). Relating spectral shape to cyanobacterial blooms in the Laurentian Great Lakes. Int. J. Remote Sens..

[16] Tomlinson M.C., Wynne T.T., Stumpf R.P. (2009). An evaluation of remote sensing techniques for enhanced detection of the toxic dinoflagellate, *Karenia brevis*. Remote Sens. Environ..

[17] Cannizzaro J.P., Carder K.L., Chen F.R., Heil C.A., Vargo G.A. (2008). A novel technique for detection of the toxic dinoflagellate, *Karenia brevis*, in the Gulf of Mexico from remotely sensed ocean color data. Cont. Shelf Res..

[18] Tao B., Mao Z., Lei H., Pan D., Shen Y., Bai Y., Zhu Q., Li Z. (2015). A novel method for discriminating *Prorocentrum donghaiense* from diatom blooms in the East China Sea using MODIS measurements. Remote Sens. Environ..

[19] Son Y.B., Kang Y.H., Ryu J.H., Jeong J.C. (2012). Monitoring red tide in South Sea of Korea (SSK) using the Geostationary Ocean Color Imager (GOCI). Korean J. Remote Sens..

[20] Lou X., Hu C. (2014). Diurnal changes of a harmful algal bloom in the East China Sea: Observations from GOCI. Remote Sens. Environ..

[21] Kim Y., Byun Y., Kim Y., Eo Y. (2009). Detection of *Cochlodinium polykrikoides* red tide based on two-stage filtering using MODIS data. Desalination.

[22] Shin J.S., Min J.E., Ryu J.-H. (2017). A study on red tide surveillance system around the Korean coastal waters using GOCI. Korean J. Remote Sens..

[23] Dierssen H.M., Kudela R.M., Ryan J.P., Zimmerman R.C. (2006). Red and black tides: Quantitative analysis of water-leaving radiance and perceived color for phytoplankton, colored dissolved organic matter, and suspended sediments. Limnol. Oceanogr..

[24] Sasaki H., Tanaka A., Iwataki M., Touke Y., Siswanto E., Tan C.K., Ishizaka J. (2008). Optical properties of the red tide in Isahaya Bay, southwestern Japan: Influence of chlorophyll a concentration. J. Oceanogr..

[25] Shin J., Kim K., Ryu J.H. (2018). Red Tide Detection through Image Fusion of GOCI and Landsat OLI. Korean J. Remote Sens..

[26] Shin J., Kim K., Son Y.B., Ryu J.H. (2019). Synergistic effect of multi-sensor Data on the detection of *Margalefidinium polykrikoides* in the South Sea of Korea. Remote Sens..

[27] Sakuno Y., Maeda A., Mori A., Ono S., Ito A. (2019). A Simple Red Tide Monitoring Method using Sentinel-2 Data for Sustainable Management of Brackish Lake Koyama-ike, Japan. Water.

[28] Khalili M.H., Hasanlou M. (2019). Harmful Algal Blooms Monitoring Using SENTINEL-2 Satellite Images. Int. Arch. Photogramm. Remote Sens. Spatial Inf. Sci..

[29] Gómez-Chova L., Tuia D., Moser G., Camps-Valls G. (2015). Multimodal classification of remote sensing images: A review and future directions. Proc. IEEE.

[30] Camps-Valls G., Tuia D., Bruzzone L., Benediktsson J.A. (2014). Advances in hyperspectral image classification: Earth monitoring with statistical learning methods. IEEE Signal Process. Mag..

[31] Gislason P.O., Benediktsson J.A., Sveinsson J.R. (2006). Random forests for land cover classification. Pattern Recogn. Lett..

[32] Muhlbauer A., McCoy I.L., Wood R. (2014). Climatology of stratocumulus cloud morphologies: Microphysical properties and radiative effects. Atmos. Chem. Phys..

[33] Landschützer P., Gruber N., Bakker D.C., Schuster U., Nakaoka S.I., Payne M.R., Sasse T., Zeng J. (2013). A neural network-based estimate of the seasonal to inter-annual variability of the Atlantic Ocean carbon sink. Biogeosciences.

[34] Zhang L.P., Zhang L.F., Du B. (2016). Deep learning for remote sensing data: A technical tutorial on the state of the art. IEEE Geosci. Remote Sens. Mag..

[35] Ball J.E., Anderson D.T., Chan C.S. (2017). Comprehensive survey of deep learning in remote sensing: Theories, tools, and challenges for the community. J. Appl. Remote Sens..

[36] Racah E., Beckham C., Maharaj T., Kahou S.E., Pal C. (2016). ExtremeWeather: A large-scale climate dataset for semisupervised detection, localization, and understanding of extreme weather events. Adv. Neural Inform. Process. Syst..

[37] Lu W., Su H., Yang X., Yan X.H. (2019). Subsurface temperature estimation from remote sensing data using a clustering-neural network method. Remote Sens. Environ..

[38] Li X., Liu B., Zheng G., Ren Y., Zhang S., Liu Y., Gao L., Liu Y., Zhang B., Wang F. (2020). Deep-learning-based information mining from ocean remote-sensing imagery. Natl. Sci. Rev..

[39] Li R., Liu W., Yang L., Sun S., Hu W., Zhang F., Li W. (2018). Deepunet: A deep fully convolutional network for pixel-level sea-land segmentation. IEEE J. Sel. Topics Appl. Earth Observ. Remote Sens..

[40] Reichstein M., Camps-Valls G., Stevens B., Jung M., Denzler J., Carvalhais N. (2019). Deep learning and process understanding for data-driven Earth system science. Nature.

[41] Cheng W., Hall L.O., Goldgof D.B., Soto I.M., Hu C. Automatic red tide detection from MODIS satellite images. Proceedings of the 2009 IEEE International Conference on Systems, Man and Cybernetics.

[42] Lee M.S., Park K.A., Chae J., Park J.E., Lee J.S., Lee J.H. (2020). Red tide detection using deep learning and high-spatial resolution optical satellite imagery. Int. J. Remote Sens..

[43] Shanmugam P., Ahn Y.H., Ram P.S. (2008). SeaWiFS sensing of hazardous algal blooms and their underlying mechanisms in shelf-slope waters of the Northwest Pacific during summer. Remote Sens. Environ..

[44] Shin J., Kim S.M., Ryu J.H. (2019). Machine learning approaches for quantifying *Margalefidinium polykrikoides* bloom from airborne hyperspectral imagery. J. Coast. Res..

[45] Kim S.M., Shin J., Baek S., Ryu J.-H. (2019). U-Net convolutional neural network model for deep red tide learning using GOCI. J. Coast. Res..

[46] Yoon H.J., Nam K.W., Cho H.G., Beun H.K. (2004). Study on monitoring and prediction for the red tide occurrence in the middle coastal area in the South Sea of Korea II. The relationship between the red tide occurrence and the oceanographic factors. J. Korea Instit. Inf. Commun. Eng..

[47] Planet (2018). Planet Imagery Product Specification. https://assets.planet.com/docs/Combined-Imagery-Product-Spec-Dec-2018.pdf.

[48] Oh S.-Y., Kim D.-H., Yoon H.-J. (2016). Application of unmanned aerial image application red tide monitoring on the aquaculture fields in the coastal waters of the South Sea, Korea. Korean J. Remote Sens..

[49] Ahn Y.H., Shanmugam P., Ryu J.-H., Jeong J.C. (2006). Satellite detection of harmful algal bloom occurrences in Korean waters. Harmful Algae.

[50] Ronneberger O., Fischer P., Brox T. U-net: Convolutional networks for biomedical image segmentation. Proceedings of the International Conference on Medical Image Computing and Computer-Assisted Intervention.

[51] Bishop C.M. (2006). Pattern Recognition and Machine Learning.

[52] Murphy K.P. (2012). Machine Learning: A Probabilistic Perspective.

[53] Kohavi R. (1998). Glossary of terms. Mach. Learn..

[54] Park J.G., Jeong M.K., Lee J.A., Cho K.J., Kwon O.S. (2001). Diurnal vertical migration of a harmful dinoflagellate, *Cochlodinium polykrikoides* (Dinophyceae), during a red tide in coastal waters of Namhae Island, Korea. Phycologia.

[55] Noh J.H., Kim W., Son S.H., Ahn J.H., Park Y.J. (2018). Remote quantification of *Cochlodinium polykrikoides* blooms occurring in the East Sea using geostationary ocean color imager (GOCI). Harmful Algae.

